# Epizootiology and biological characteristics of echinococcosis in agricultural animals, dogs, wild carnivores, and rodents in the Western region of the Republic of Kazakhstan

**DOI:** 10.14202/vetworld.2023.2277-2286

**Published:** 2023-11-12

**Authors:** Abirova Ilana, Baitlesov Erbulat Upievich, Kereyev Abzal Kenesovich, Mamanova Saltanat Bekbosynovna, Zakirova Faruza Bakitzhanovna, Murzabaev Kenzhebek Esmagambetovich, Sengaliyev Yerbol Maratovich, Satybaev Berik Garipullievich, Abdrakhmanov Rinat Gabdullinovich

**Affiliations:** 1 Department of Veterinary Medicine and Animal Husbandry West Kazakhstan Agrarian and Technical University Named after Zhangir Khan, Uralsk 090009, Republic of Kazakhstan; 2 Department of Veterinary Medicine and Technosphere Safety, West Kazakhstan Innovative and Technological University, Uralsk 090009, Republic of Kazakhstan; 3 Laboratory of Virology, Kazakh Scientific Research Veterinary Institute, Almaty 050016, Republic of Kazakhstan

**Keywords:** agricultural animals, dogs, *Echinococcus*, Kazakhstan, rodents, wild carnivores

## Abstract

**Background and Aim:**

Echinococcosis is one of the most dangerous parasitic diseases common to humans and animals. In Kazakhstan, echinococcosis is widespread in animals. This study aimed to estimate the prevalence and biological characteristics of echinococcosis in agricultural animals, dogs, wild carnivores, and rodents in the Western region of the Republic of Kazakhstan.

**Materials and Methods:**

The study analyzed slaughtered carcasses of cattle (2500), sheep (4200), pigs (250), horses (91), and camels (45). Furthermore, the study analyzed 144 dogs (herding, rural, and urban), 41 wild carnivores (wolves, red foxes, and corsac foxes), and 339 wild rodents (great gerbils, tamarisk jirds, little ground squirrels, yellow ground squirrels, and muskrats). Postmortem and vital examination methods were used in the helminthological studies of dogs, wild carnivores, and rodents. In agricultural animals, localization and intensity were determined by counting echinococcal cysts in parenchymatous organs.

**Results:**

Extensiveness of invasion (EI) averaged 19.2% in cattle, 27.5% in sheep, 5.6% in pigs, and 13.3% in camels. Echinococcal cysts mainly affected the liver in sheep (45.4%) and the lungs in cattle (35.5%). The fertility of echinococcal cysts decreased with age in animals. Acephalocysts were registered mainly in cattle. The highest invasiveness of *Echinococcus* *granulosus* was found in herding dogs with an EI of 12.5%. Experimental infestations of dogs showed that maturation of echinococcal eggs occurred by days 36–40 and maturation of segments by day 50. Studies of wild carnivores and rodents showed the presence of *E. granulosus* (imago stage) in wolves, *Alveococcus multilocularis* (imago stage) in red foxes and corsac foxes, and *A. multilocularis* (larval stage) in great gerbils and muskrats.

**Conclusion:**

Our data provide evidence of high epizootiological danger for the population and significant damage caused by *E. granulosus* to animal husbandry in the region. Studies on the spread of echinococcosis suggest the possibility of controlling the situation with human and animal diseases and show the importance of this issue.

## Introduction

Echinococcosis has global significance in veterinary and medical parasitology. Echinococcosis is a type of biohelminthiasis that occurs in domestic and wild animals and humans. The helminth circulates in the natural biocoenosis with high extensiveness of invasion (EI), as determined by the presence of all members of the epizootic chain. Most literary sources indicate that a definitive host is the source of invasion, although both definitive and intermediate hosts may be sources of invasion. The parasite mainly infects ruminants in almost all countries of the world, causing significant economic damage to farms. Echinococcosis is a chronic helminthiasis that develops with a change of hosts. In the sexually mature stage, the helminth parasitizes the small intestine of carnivores. Larvocysts grow in the tissues and organs of animals and humans. Dogs are the main source of infection for farm animals and humans, and the abiotic environment containing *Echinococcus* oncospheres is the transmission factor for animals. Oncospheres are hematogenously spread throughout the body but are more often localized in the liver and lungs [1, 2].

The Western region of the Republic of Kazakhstan is predominantly cattle-breeding. The livestock complex consists mainly of cattle and sheep breeding. In addition, the region contains many wild animals.

Monitoring the epizootic situation of echinococcosis comprises studying data from many animal species. Epizootiological surveillance of the teniids is marked by certain features. The life cycles of biohelminths are much more complex than those of microbes and protozoa, and in many species, are associated with changes in developmental stages and habitats. Therefore, to assess the risk of infection, epizootiological and epidemiological conditions, and animal morbidity, it is necessary to use data on the presence of intermediate and additional hosts and lesions of helminths (larval stage). The combined foci of various parasitic diseases in the same area are due to the presence of common hosts of agents. This, in turn, determines the risk of infection for animals and humans. Combined foci leads to panzootic diseases, indicating the need for an integrated approach to monitoring echinococcosis [3–5].

This study aimed to evaluate the epizootiological role of carnivores and small mammals as reservoirs of pathogens that cause dangerous diseases in humans and farm animals in the Western region of the Republic of Kazakhstan. The findings of this study provide insights into the control of human and animal diseases.

## Materials and Methods

### Ethical approval

All procedures were discussed and approved at the meetings of the local biological ethics committee of the Zhangir Khan West Kazakhstan Agrarian and Technical University of the Republic of Kazakhstan (Approval number: ZKATU-1/2017 of February 23, 2017).

### Study period and location

The study was conducted from March to December 2017 at the West Kazakhstan Veterinary Science Research Station, the Zhangir Khan West Kazakhstan Agrarian and Technical University, farms, meat packing plants, and slaughterhouses in the Western Region. The distribution and age dynamics, fertility, and localization of echinococcosis in farm animals were determined based on autopsies of animals of different ages. In total, the study involved the examination of slaughtered carcasses from cattle (2500), sheep (4200), pigs (250), horses (91), and camels (45). Furthermore, 144 dogs, 41 wild carnivores, and 339 rodents were inspected.

### Sample collection

#### Localization and morphological characteristics of larval echinococcosis in farm animals

The localization and intensity of invasions (II) were determined by counting echinococcal cysts in the lungs, liver, kidneys, heart, and spleen. Animals were divided into three groups according to degree of invasion: weak: 5, moderate: 6–10, and severe: >10 echinococcal cysts per animal. The organs were examined using palpation and incisions. Cyst size was measured using a caliper.

#### Helminthological examinations of dogs and wild animals (carnivores and rodents)

Helminthological studies of dogs and wild carnivores were conducted throughout the examined territory. Postmortem methods of investigation (complete and incomplete helminthological dissection of the gastrointestinal tract) and vital methods (diagnostic deworming with arecoline hydrobromide with complete emptying of gastrointestinal contents) were used.

Unscheduled deworming with arecoline hydrobromide and autopsies was performed to evaluate the total number and species composition of helminths in dogs. Diagnostic deworming is characterized by quickness and is easily applicable in mass examinations of dogs. Diagnostic deworming and autopsies were performed on 144 dogs of various types, including 64 herding dogs, 36 rural dogs, and 44 urban dogs.

The intestinal localization of *Echinococcus* was examined in six experimentally infected dogs (age from 6 months to 5 years) at different times, starting from days 15 to 50 after exposure. The first experiment included three 6-month-old pups. The animals were infected with protoscoleces taken from the echinococcal cysts of sheep liver at a dose of 1500 pcs. Viability was checked by heating. The pups were examined on days 15, 30, and 36 after infection. In the second experiment, three dogs were fed 1000 protoscoleces each. The dogs were examined on days 30, 40, and 50 after infection. All experimental infections were conducted according to the principles of the good laboratory practice standard, considering the European Convention for the Protection of Vertebrate Animals Used for Experiments or Other Scientific Purposes and the Guide for the Care and Use of Laboratory Animals (2010). Experimental infections were performed in the Laboratory of Biological Safety of the Department of Epizootiology, Parasitology, and Veterinary and Sanitary Expertise of Zhangir Khan West Kazakhstan Agrarian-Technical University.

To determine the species composition of helminths in the gastrointestinal tract of animals, we conducted autopsies of 20 wolves *(Canis lupus* L.), 14 red foxes *(Vulpes vulpes* L.), 7 corsac foxes (*Vulpes corsac* L.), 175 great gerbils (*Rhombomys opimus* Licht.), 90 tamarisk jirds (*Meriones tamariscinus* Pall.), 35 little ground squirrels (*Citellus pygmaeus* Pall.), 30 yellow ground squirrels (*Citellus fulvius* Licht.), and 9 muskrats (*Ondatra zibethica* L.).

Examination of wild carnivores, specifically wolves, red foxes, and corsac foxes, was performed after they had been shot and in fallen animals. The organs, including the intestines of wild animals were delivered by hunters, gamekeepers, and veterinary inspectors. If necessary, internal organs were preserved in 3% formalin solution. Rodents were captured using standard pitfall traps. Helminths species were established with the help of “Atlas of the most widespread helminths in agricultural animals” by VF Kapustin and “Identifier of helminths of rodents of the fauna” [6–9].

### Statistical analysis

Statistical analysis of numerical data was performed using Microsoft Excel 2010 (Microsoft Corp., Washington, USA). Quantitative measures of invasion were EI expressed as a percentage and invasion intensity (II).

## Results

### Incidence of echinococcosis in farm animals in the Western region

Domestic animals played a leading role in the spread of larval echinococcosis in the Western region. Infestation averaged 19.2% for cattle, 27.5% for sheep, 5.6% for pigs, and 13.3% for camels (Table-1).

**Table-1:** Incidence of echinococcosis in farm animals.

Animal species	Heads examined	Of these infested	EI, %
Cattle	2500	480	19.2
Sheep	4200	1155	27.5
Pigs	250	14	5.6
Horses	91	-	-
Camels	45	6	13.3

EI=Extensiveness of invasion

### Localization and morphological characteristics of larval echinococcosis in cattle and sheep

**Figure-1:**
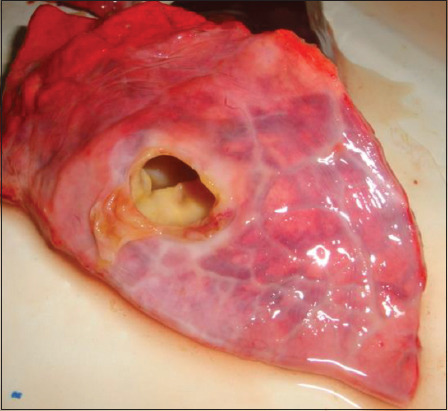
Echinococcosis of the lung of cattle.

Morphological modification of echinococcal cysts was examined in sheep and cattle. The internal organs (parenchymatous) of 400 cattle were examined. In cattle, the lungs (35.5%) (Figure-1) and liver (23%) were more frequently affected: simultaneous invasion of both organs was observed in 135 heads (33.75%); simultaneous invasion of the kidneys, heart, and spleen was found in 29 heads (7.25%). Severe liver damage was observed in 27.17%, moderate in 40.21%, and weak in 39.13%. A high degree of lung damage was observed in 26.05%, moderate in 27.46%, and weak in 45.07% (Table-2).

**Figure-2:**
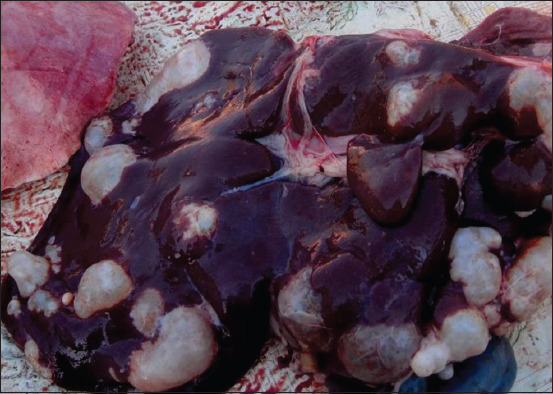
Echinococcosis of sheep liver.

**Table-2:** Invasion intensity (II) and localization of echinococcal cysts in the organs of cattle.

Total number of examined	Including		II					
	
	Number of organs affected	%	Weak	%	Moderate	%	Severe	%
400	Liver 92	23	36	39.13	37	40.21	25	27.17
	Lungs 142	35.5	64	45.07	39	27.46	37	26.05

The internal organs of 1100 sheep were examined for echinococcal infection. The liver was affected in 500 sheep (45.4%) (Figure-2); lungs in 400 sheep (36.3%); and heart, kidneys, and spleen in 40 sheep (3.63%). Simultaneous liver and lung lesions were found in 160 sheep (14.54%). Severe liver damage was observed in 24.0%, moderate in 30.0%, and weak in 46.0%. Severe lung damage was found in 38.25%, moderate in 33.75%, and weak in 28% (Table-3).

**Table-3:** Invasion intensity (II) a nd localization of echinococcal cysts in the organs of sheep.

Total number of examined	Including		II					
	
	Number of organs affected	%	Weak	%	Moderate	%	Severe	%
1100	Liver 500	45.4	230	46.0	150	30.0	120	24.0
	Lungs 400	36.3	112	28.0	135	33.75	153	38.25

**Figure-3:**
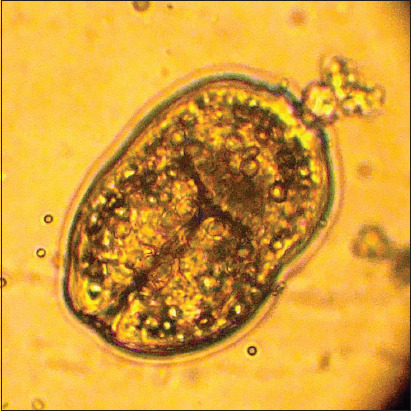
Physiological condition of *Echinococcus* cysts (cephalocysts protoscolex).

**Figure-4:**
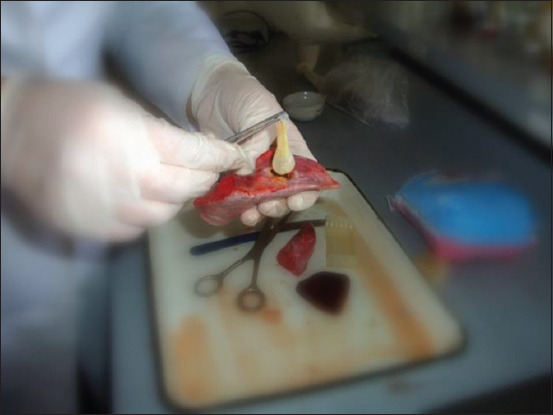
Physiologic condition of *Echinococcus* cysts (cephalocysts).

The investigation of the physiological state of *Echinococcus granulosus* larval cysts from different animal species allowed us to determine their significance in the epizootiology of echinococcosis. We examined larval cysts from different age groups of cattle and sheep for protoscoleces (Figure-3). Data analysis of echinococcal larval cysts (Figure-4) in Table-4 shows that the number of fertile larval cysts decreases with age in cattle.

**Table-4:** Physiological condition of echinococcal cysts detected in cattle.

Age of cattle	Cysts examined (pcs.)	Condition of cysts					

		Cephalocysts		Acephalocysts		Petrified	
		
		n	%	n	%	n	%
Under 3 years	38	8	21.05	16	42.1	14	36.84
3–5 years	130	42	32.3	81	62.3	7	5.38
Over 5 years	82	2	2.43	64	78.04	16	19.51

Only acephalocysts (45.0%) and petrified cysts (55.0%) were detected in animals up to 1 year of age. A greater number of cephalocysts were observed in animals aged 2–5 years: 20.69%–56.10% (Table-5). In older animals, the number of cephalocysts decreased and the number of acephalocysts or petrified cysts increased.

**Table-5:** Physiological condition of echinococcal cysts detected in sheep.

Age of sheep	Cysts examined (pcs.)	Condition of cysts					

		Cephalocysts		Acephalocysts		Petrified	
		
		n	%	n	%	n	%
Under 1 year	20	0	-	9	45.0	11	55.0
2 years	58	12	20.69	18	31.03	28	48.28
3 years	120	65	54.17	35	29.17	20	16.66
4 years	205	115	56.10	50	24.39	40	19.51
5 years	200	65	32.5	80	40.0	55	27.5
Over 5 years	320	101	31.56	94	29.38	125	39.06

### Invasion of dogs with echinococcosis and other intestinal helminths

*Taenia hydatigena* invasion was the greatest in rural (15.63%) and herding dogs (16.67%); urban dogs were less infested at 14.55%. Rural (8.33%) and herding dogs (7.81%) were most infested with *Multiceps multiceps*. Comparatively, urban dogs were less infested at 2.27%. The greatest invasiveness of *E. granulosus* was observed in herding dogs (EI = 12.5%), whereas in urban and herding dogs, EI was 4.55% and 11.11%, respectively. High EI of *Dipylidium caninum* was observed in urban dogs (20.45%). This value was much higher than that in rural and herding dogs, with EI 8.33% and 6.25%, respectively (Table-6).

**Table-6:** Extensiveness of helminth invasion in dogs in the Western Region according to diagnostic deworming and autopsy data.

S. No.	Helminth species	Total, 144 dogs, EI, %	Herding, 64 dogs, EI, %	Rural, 36 dogs, EI, %	Urban, 44 dogs, EI, %
1.	*Taenia hydatigena*	12.5	15.63	16.67	4.55
2.	*Multiceps multiceps*	6.25	7.81	8.33	2.27
3.	*Echinococcus granulosus*	9.72	12.5	11.11	4.55
4.	*Dipylidium caninum*	11.11	6.25	8.33	20.45
5.	*Toxocara canis*	17.36	17.19	19.44	15.91
6.	*Toxascaris leonina*	11.11	14.06	11.11	6.82
7.	*Uncinaria stenocephala*	3.47	2.27	4.54	9.09

EI=Extensiveness of invasion

Overall, the highest indicator of invasion by *Toxocara canis* was demonstrated mainly by rural and herding dogs at 19.44% and 17.19%, respectively. The rate of urban dogs (15.91%) was the lowest and more similar to the indicators of herding and rural dogs.

Invasion by *Toxascaris leonina* was the lowest among urban dogs (6.82%). Rural and herding dogs showed higher percentages at 11.11% and 14.06%, respectively. The highest share of invasions by *Uncinaria stenocephala* was observed in urban dogs. These nematodes were found exclusively in urban areas, and only a small proportion of infested dogs was found among rural and herding dogs. Urban dogs had the highest level of *U. stenocephala* invasion (9.09%), followed by rural (4.54%) and herding (2.27%) dogs.

### Localization of *Echinococcus* in dog intestine

In the pup examined 15 days after infection, echinococci were found in the anterior third of the intestine. In the second pup, dissected on day 30, the anterior third of the intestine contained 300 echinococci. The echinococci had two segments. The length of echinococci was 2–5 mm. On day 36 after infection, the feces of the third pup contained segments containing *Echinococcus* eggs. After slaughter, >400 echinococci were observed in the jejunum, having segments with formed eggs.

After 30 days of infection, the first dissected dog (6 years old) had individual specimens in the duodenum and the midgut and a small number in the rest of the small intestine. A total of 310 echinococci were found (1.5–4 mm in length). The strobilus had one and two segments, with eggs absent. Autopsy of the second dog was performed on day 40 after infection, revealing 85 echinococci, 3–5 mm long, with two and three segments in the middle part of the small intestine, and some echinococci with eggs in the third segment. In the third dog examined on day 50, 72 echinococci (4–6.5-mm long) were found in the jejunum, and isolated specimens were present throughout the small intestine. The cestodes had 2–4 segments, and many eggs were found in the latter. Fecal examinations performed before slaughter on day 50 showed the beginning of egg release.

### Invasion of wild animals (carnivores and rodents) with echinococcosis and other intestinal helminths

#### Wild carnivores

Below we present the results of the examination of animals of each species with the indication of their number and infestation with zoonotic helminths (Table-7). To understand the epizootiological situation with *E. granulosus* and *Alveococcus multilocularis*, we only examined fragments of the gastrointestinal tract of wild carnivores.

**Table-7:** Results of helminthological studies of wild carnivores.

S. No.	Helminth species	Animal species					

		Wolf, n-20		Red fox, n-14		Corsac fox, n-7	
		
		EI, %	II, pcs.	EI, %	II, pcs.	EI, %	II, pcs.
1.	*Echinococcus granulosus*	35.0	90–1,500	-	-	-	-
2.	*Alveococcus multilocularis*	-	-	28.57	25–90	28.57	25–70
3.	*Taenia hydatigena*	25.0	2–8	-	-	-	-
4.	*Taenia pisiformis*	5.0	4–7	-	-	-	--
5.	*Taenia crassiceps*	-	-	7.14	1–3	-	-
6.	*Dipylidium caninum*	45.0	4–27	51.14	3–15	100	8–15
7.	*Toxocara canis*	40.0	6–50	21.43	6–8	28.57	5–7
8.	*Toxascaris leonina*	30.0	4–25	35.71	5–12	-	-
9.	*Moniliformis moniliformis*	-	-	7.14	3–6	-	-

EI=Extensiveness of invasion, II=Invasion intensity

Wolf (*C. lupus* L.)

**Figure-5:**
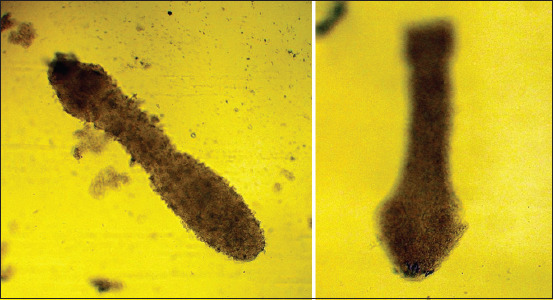
Sexually mature *Echinococcus granulosus*.

Infestation was 100% with different helminth species. Of the 20 studied wolves, sexually mature *E. granulosus* was found in 7 (35.0%) (Figure-5). The number of detected helminths per wolf ranged from 90 to 1500 pcs. *Dipylidium caninum* was found in 9 (45.0%) wolves (II = 4–27 pcs.). *Taenia hydatigena* was detected in 5 wolves (25.0%) (II = 2–8 pcs.). Two *Taenia pisiformis* cysts were found in 1 (5.0%) wolf. *Toxascaris leonina* was detected in 6 (30.0%) wolves (II = 4–25 pcs.). *Toxocara canis* was present in 8 (40.0%) wolves (II = 6–50 pcs.).

Red fox (*V. vulpes* L.)

Out of 14 red foxes, *A. multilocularis* was detected in 4 (28.57%) (II = 25–90 pcs.). *Dipylidium caninum* was found in 8 foxes (51.14%) (II = 3–15 pcs.). *Toxascaris leonina* was found in 5 (35.71%) foxes (II = 5–12 pcs.), and *T. canis* was found in 3 (21.42%) foxes (II = 6–8 pcs.). *Moniliformis moniliformis* was detected in 1 fox (7.14%) (II = 3–6 pcs.). *Taenia crassiceps* occurred in 1 (7.14%) fox (II = 1–3 pcs.). Thus, invasion by different helminth species totaled 100%.

Corsac fox (*V. corsac* L.)

Three helminth species were detected in 7 corsac foxes examined. *Alveococcus multilocularis* was detected in 2 corsac foxes (28.57%) (II = 25–70 pcs.). All of the examined corsac foxes were infected with *D. caninum* (II = 8–15 pcs.). *Toxocara canis* was found in 2 (28.57%) foxes (II = 5–7 pcs.). Thus, infestation with different helminth species totaled 100%.

#### Wild rodents

Great gerbil (*R. opimus* Licht.)

Of the 175 great gerbils studied, 103 (58.85%) were infected with helminths. *Alveococcus multilocularis* was discovered in 22 great gerbils (12.57%) (II = 3–7 pcs.). *Trichocephalus rhombomidi*s was found in 28 (16.0%) great gerbils (II = 1–12 pcs.). *Dentostomella translucida* was identified in 15 great gerbils (8.57%) (II = 3–6 pcs.). *Aspiculuris asiatica* infested 38 (21.71%) rodents (II = 1–90 pcs.).

Tamarisk jird (*M. tamariscinus* Pall.)

Of the 90 jirds studied, 58 (64.44%) were infected with various species of helminths. *Multiceps endothoracicus* was found in 14 (15.55%) individuals (II = 8–12 pcs.). *Rodentotaenia merionidis* was detected in 8 (8.88%) jirds (II = 2–9 pcs.). *Mastophorus muris* was detected in 36 jirds (40.0%) (II = 2–4 pcs.).

Little ground squirrel (*C. pygmaeus* Pall.)

All 35 little ground squirrels studied were infected with helminths (100%). The EI of *Streptopharagus rutassi* was 29 (82.85%) (II = 2–7 pcs.). *Rictularia caucasica* parasitized 4 (11.42%) (II = 5–6 pcs.). *Moniliformis moniliformis* was found in 2 (5.71%) animals (II = 1–2 pcs.).

Yellow ground squirrel (*C. fulvus* Licht.)

Among the 30 rodents dissected, 13 were infected with helminths (43.33%). *Taenia tenuicollis* was found in 10 (33.33%) individuals (II = 1–2 larvae). *Moniliformis moniliformis* Acanthocephala was found in 3 (10.0%) yellow ground squirrels (II = 1–2 pcs.).

Muskrat *(O. zibethica* L.)

On dissection, it was observed that all nine muskrats (100%) were infected with helminths. Of them, 4 (44.44%) were infected with *Plagiorchis elegans* (II = 8–15 pcs.). *Quingueserialis quingueserialis* infected 2 (22.22%) animals (II = 5–20 pcs.). *Alveococcus multilocularis* infected 3 (33.33%) muskrats (II = 2–8 cysts) (Table-8).

**Table-8:** Results of helminthological studies of rodents.

S. No.	Helminth species	Animal species									

		Great gerbil, n-175		Tamarisk jird, n-90		Little ground squirrel, n-35		Yellow ground squirrel, n-30		Muskrat, n-9	
				
		EI, %	II, pcs.	EI, %	II, pcs.	EI, %	II, pcs.	EI, %	II, pcs.	EI, %	II, pcs.
1	*Alveococcus multilocularis*	12.57	3–7	-	-	-	-	-	-	33.33	2–8
2	*Multiceps endothoracicus*	-	-	15.55	8–12	-	-	-	-	-	-
3	*Rodentotaenia merionidis*	-	-	8.89	2–9	-	-			-	-
4	*Taenia tenuicollis*	-	-	-	-	-	-	33.33	1–2	-	-
5	*Trichocephalus rhombomidis*	16.0	1–12	-	-	-	-	-	-	-	-
6	*Dentostomella translucida*	8.57	3–6	-	-	-	-	-	-	-	-
7	*Aspiculuris asiatica*	21.71	1–90	-	-	-	-	-	-	-	-
8	*Mastophorus muris*	-	-	40.0	2–4	-	-	-	-	-	-
9	*Streptopharagus rutassi*	-	-	-	-	82.85	2–7	-	-	-	-
10	*Rictularia caucasica*	-	-	-	-	11.42	5–6	-	-	-	-
11	*Plagiorchis elegans*	-	-	-	-			-	-	44.44	8–15
12	*Quingueserialis quingueserialis*	-	-	-	-			-	-	22.22	5–20
13	*Moniliformis moniliformis*	-	-	-	-	5.71	1–2	10.0	1–2	-	-

EI=Extensiveness of invasion, II=Invasion intensity

## Discussion

### Incidence of echinococcosis in agricultural animals in the Western region

Despite the high level of veterinary medicine available, the issue of parasitic diseases, particularly echinococcosis of farm animals, remains urgent. Echinococcosis is registered in all parts of the world. This testifies to the high level of invasion of agricultural animals with echinococcosis and the wide spread of echinococcosis. The Western region is largely cattle-breeding. The livestock complex consists mainly of cattle and sheep breeding. Domestic animals play a leading role in the dissemination of larval echinococcosis in the Western region. Infestation averages 19.2% in cattle, 27.5% in sheep, 5.6% in pigs, and 13.3% in camels [10].

### Localization and morphological characteristics of larval echinococcosis in cattle and sheep

The ecology and epizootiology of echinococcosis directly depend on the age and species of the animal, localization, intensity, and fertility. The high fertility of *Echinococcus* allows it to retain a large population size in the body of definitive and intermediate hosts, thereby maintaining a continuous epizootic chain. To maintain the epizootic chain, the factors of preservation and transmission of infestation must be biologically active. Such factors include echinococcal cysts, with a well-developed germinal shell and viable protoscoleces.

We found different sizes of collapsed shells of maternal cysts, squeezed by growing daughter cysts in echinococcal cysts taken from the parenchymatous organs of cattle (age ≥5 years). Endogenous formation of daughter cysts was observed only when maternal cysts were damaged and decayed. Maternal cyst shells were characterized by jaundice, transparency, and varying thickness. Daughter cysts were distinguished by elasticity and spherical shape and varied in size from 1 to 9 cm.

A high percentage of fertile cysts was observed in sheep, and lower percentages were found in cattle. Sheep aged 2–5 years in natural conditions had the greatest epizootic value, and therefore, are the primary link in the persistence of invasion and parasitization.

In all animal species, the intensity of infestation increased with age; the liver (45.4%) in sheep and the lungs (35.5%) in cattle were the most affected. The fertility of echinococcal cysts decreases with age in animals [11].

### Invasion of dogs with echinococcosis and other intestinal helminths

Studies have reported that parasitic systems function in the canine population, with >20 helminth species as coactants, most of which parasitize the gastrointestinal tract of animals in the sexually mature state. Helminths are detrimental to the health of dogs and contaminate the environment, thus creating the preconditions for a parasitological pandemic in the territory. Dogs pose a considerable epidemic danger to residents in both urban and rural areas because they are hosts to several helminths dangerous to humans. This threat is multiplied by the large number of stray animals.

In all groups of dogs by economic use in the region, we observed four species of the cestode (*T. hydatigena*, *M. multiceps*, *E. granulosus*, and *D. caninum*); from the family *Dipylidiidae (D. caninum*); from nematodes two species of ascarids (*T. canis* and *T. leonine*), and one species of Ancylostomatidae (*U. stenocephala*). All dogs had helminths in the small intestine.

Our research established that seven helminth species circulate in dogs in the Western region, including four cestode and three nematode species. Based on the dynamics of helminth infestation in dogs, we determined the main factors influencing the presence and number of infestations.

In all farms, dogs were either free-roaming or leashed. The farms lack well-equipped collection and disposal locations for livestock waste and carcasses of fallen animals. Moreover, the by-products of meat and meat confiscated from slaughterhouses are fed to dogs. The situation is aggravated by the rapidly developing private livestock sector and its lack of veterinary and sanitary measures. Private farms try to avoid the necessary veterinary treatments to save money. On farms, dogs are trained to deal with livestock and kept with them for herd protection. In addition, the uncontrolled breeding of dogs and their infestation with teniids contaminates the area with infestation elements, creating new opportunities for the infestation of agricultural animals [12].

### Localization of *Echinococcus* in the intestines of dogs

Our studies conducted on dogs aged 6 months to 5 years identified specific features of the localization of different stages of *Echinococcus* in the intestine of the definitive host. Our data show that the maturation of echinococcal eggs occurs on days 36–40 and the maturation of segments occurs on day 50. Echinococci were unevenly distributed in the intestine of dogs, isolated specimens were found in the duodenum, and the largest number was found in the middle part of the small intestine [13].

### Invasion of wild animals (carnivores and rodents) with echinococcosis and other intestinal helminths

#### Wild carnivores

Wolf (*C. lupus* L.)

Helminthological examination of the gastrointestinal tract of wolves revealed that they were infected with different species of zoonotic helminths. During autopsy of the gastrointestinal tract of 20 wolves collected from different areas of the Western region, six helminth species were identified, including four species of cestodes (*E. granulosus*, *D. caninum*, *T. hydatigena*, and *T. pisiformis*) and two species of nematodes (*T. canis* and *T. leonina)*. *E. granulosus* was localized in the middle part of the jejunum. *Taenia hydatigena* was localized along the entire length of the jejunum. *Taenia pisiformis* and *T. canis* were localized in the small intestine. *Dipylidium caninum* was localized at the beginning of the ileum. *Toxascaris leonina* was localized in the duodenum and jejunum. Echinococci most often parasitize in combination with *T. hydatigena* and *D. caninum*. Monoinvasion was noted in 3 (15.0%) animals; the first had *E. granulosus* (4600 pcs.), the second had *D. caninum*, and the third had *T. canis*. Polyinvasion was determined in 17 (85.0%) animals. Polyinvasion was most often represented by a combination of *T. hydatigena* and *E. granulosus* [14].

Red fox (*V. vulpes* L.)

Among the examined foxes, 8 (51.14%) were infected with various species of helminths. We identified three cestode species (*A. multilocularis*, *D. caninum*, and *T. crassiceps*), two nematode species (*T. leonina* and *T. canis*), and one Acanthocephala species (*M. moniliformis*). *Alveococcus multilocularis* was localized in the middle part of the jejunum. *Dipylidium caninum* and *M. moniliformis* were localized at the beginning of the ileum. *Toxascaris leonina* and *T. canis* were localized in the small intestine. *Taenia crassiceps* was localized in the jejunum. Monoinvasion was observed in 3 (21.42%) foxes and polyinvasion in 11 (78.57%). Two helminth species, *D. caninum* and *T. canis* were parasitized as monoinvasion. Polyinvasion was recorded as a combination of *T. canis* with *M. moniliformis* and *A. multilocularis* with *D. caninum* [15].

Corsac fox (*V. corsac* L.)

Helminthological studies of corsac foxes identified three species of helminths, including two cestode (*A. multilocularis* and *D. caninum*) and one nematode (*T. canis*) species. *Alveococcus multilocularis* was localized in the middle part of the jejunum. *Dipylidium caninum* and *T. canis* were localized in the small intestine. Monoinvasion was observed in 1 (14.28%) case with *A. multilocularis*, and polyinvasion was recorded in 6 (85.71%) animals. Polyinvasion was most often represented by combinations of *T. canis–D. caninum* and *T. canis–D. caninum–A. multilocularis* [16].

The presence of *A. multilocularis* in the helminth fauna of these predators indicates the specificity of their trophic relationships in local conditions and high availability. In general, the species composition of helminths in these predators, as well as dogs, in the region was relatively monotypic. Due to a rather small number of helminthological studies, we managed to not only obtain interesting faunistic data but also conducted a mini-monitoring of individual infestations. Although the small volume of the studied samples does not allow us to begin the investigation of the parasitic systems of individual helminths of these predators, based on the obtained data, we can establish the most general characteristics of some.

#### Wild rodents

Great gerbil (*R. opimus* Licht.)

In great gerbils, we discovered four helminth species: One cestode (*A. multilocularis*) and three nematode (*T. rhombomidis*, *D. translucida*, and *A. asiatica*) species. *Alveococcus multilocularis* was localized in the liver of great gerbils. *Trichocephalus rhombomidis* was localized in the cecum. *Dentostomella translucida* was localized in the large intestine. *Aspiculuris asiatica* was localized in the large intestine. Monoinvasion was present in 18 (10.28%). Polyinvasion was observed in combination with *A. multilocularis* and was detected in 77 (44.0%). Most often, polyinvasion occurred as a combination of *A. multilocularis* and *A. asiatica*.

Tamarisk jird (*M. tamariscinus* Pall.)

Three species of helminths were found in jirds, specifically two species of cestodes (*R. merionidis* and *M. endothoracicus*) and one of nematodes (*M. muris*). *Multiceps endothoracicus*, *R. merionidis*, and *M. muris* were localized in the thoracic cavity, whole perimeter of the small intestine, and thoracic cavity, respectively. Monoinvasion was observed in three cases (3.33%) of *R. merionidis*, and combined invasions were found in 55 (61.11%).

Little ground squirrel (*C. pygmaeus* Pall.)

Two species of nematodes (*S. rutassi* and *R. caucasica*) and one species of Acanthocephala (*M. moniliformis*) were found in little ground squirrels. *Streptopharagus rutassi*, *R. caucasica*, and *M. moniliformis* were localized in the stomach, middle one-third of the small intestine, and small intestine, respectively. Monoinvasion was not observed, but polyinvasion (100%) circulated as combinations of *M. moniliformis–R. caucasica* and *S. rutassi–R. caucasica*.

Yellow ground squirrel (*C. fulvus* Licht.)

Yellow ground squirrels had one cestode species (*T. tenuicollis*) and one Acanthocephala species (*M. moniliformis*). *Taenia tenuicollis* and *M. moniliformis* were localized in the liver and small intestine, respectively. Polyinvasion was present in six cases (20.0%) as a combination of *M. moniliformis* and *T. tenuicollis*.

Muskrat *(O. zibethica* L.)

In muskrats, we detected three species of parasitic helminths: one cestode (*A. multilocularis*) and two trematode (*P. elegans* and *Q. quingueserialis*) species. *Alveococcus multilocularis*, *P. elegans*, and *Q. quingueserialis* were localized in the liver, small intestine, and colon, respectively. Polyinvasion has been observed in all muskrats [17–22].

Thus, we discovered sexually mature and larval forms of *Echinococcus* and *Alveococcus* in the following animal species: *Echinococcus granulosus* (imago stage) in the wolf; *A. multilocularis* (imago stage) in the red fox and the corsac fox; and *A. multilocularis* (larval stage) in the great gerbil and the muskrat.

Analyzing the obtained data, we can assert that the above representatives of wild fauna often carry echinococcosis and alveococcosis infestations. Many species of wild animals inhabit natural biotopes. Individual members can play a direct role. Helminths as components of biocenoses play a significant role in the transmission of invasive diseases. Wild carnivores often reserve infestation from domestic animals. Under natural conditions, infestation of predators occurs in areas where farm animals graze. Many helminths of wild carnivores parasitize domestic animals and pose a great epidemiological hazard to the population. Many wild carnivores are the definitive hosts of *Echinococcus*. Floodplains serve as accumulators of zoonotic helminth eggs; the intensity of infestation increases when water in rivers recedes and wild carnivores go far to the banks in search of water, contaminating it with feces. In addition, rodents, which often fall prey to carnivores, play a significant role in the circulation of helminths. Rodents and carnivores share common types of diseases and together participate in the life cycle of some helminth species. Hunting in the region is one of the main factors of entry of parasitic zoonoses from the natural biocenosis into the synanthropic one.

## Conclusion

Our data demonstrate the high epizootiological and epidemiological danger posed to the population, as well as the significant damage caused by echinococcosis to livestock breeding in the region. Studies on the spread of echinococcosis suggest the possibility of monitoring the situation with the disease in humans and animals and demonstrate the importance of this issue. The results of echinococcosis monitoring provide a better understanding of the main factors related to the transmission of this parasite between animals with different habitats and further contribute to the development of effective methods for controlling this disease. Given the danger of echinococcosis to humans and animals, there is a need for continuous monitoring and a series of preventive measures to address this issue. In our study, we did not perform molecular tests for morphological identification; however, we plan to perform molecular tests for in-depth investigation of echinococcosis and other dangerous diseases in humans and farm animals.

## Authors’ Contributions

AI and BEU: Designed the study and analyzed the data. AI, SYM, SBG, and ARG: Conducted the study. ZFB and MSB: Recorded and analyzed the data. MKE and KAK: Coordinated the study and drafted and revised the manuscript. All authors have read, reviewed, and approved the final manuscript.
